# Facts about Hospitals

**Published:** 1887-06-18

**Authors:** Henry C. Burdett


### FACTS ABOUT HOSPITALS; FOR PREACHERS, ETC.

By Henry C. Burdett.

**Hospitals as they were.**

One of the most famous hospitals in the world, the Hôtel Dieu, formerly presented a truly horrible spectacle, as recorded by Bally, Tenon, and Lavoisier. Owing to want of space the general arrangement of the Hôtel Dieu was to place a great number of beds in the wards, and as many as four, five, or nine people in each bed—the dead were mingled with the living. In rooms where the passages were narrow the air became stagnant, light penetrated feebly, and patients had to go partially clad to the bridge of St. Charles to breathe the fresh air. The convalescent ward was on the third storey, and could only be reached through a room devoted to small-pox patients. The room for the insane was next to one filled with sufferers who had recently undergone the most painful operations, and who were constantly disturbed by the cries of their neighbours. The same ward often contained contagious as well as other diseases. Women suffering from small-pox were put with fever patients. The operating-room, where all kinds of operations were carried on, contained not only those who were actually undergoing operations, but also those waiting to be operated upon, and those who had already been subjected to the surgeon's knife. The operations were performed in the middle of the room; the preparations were made in sight of all the patients; the cries of those operated upon were heard by all present, terrifying those who were awaiting operation, and reviving in those already operated upon the pains they had gone through. The historian adds, "It can be well imagined how much the patients must have been affected by these heart-rending cries, these terrors, and how much it interfered with their progress to health, nay, in many instances, it may actually have endangered their lives."

**Hospitals as they are.**

What a contrast to the Hôtel Dieu is the modern metropolitan hospital! Who can praise it too highly, or describe all its beneficent work briefly and fully? It is a home of care and cure for the poor in the hour of sickness. A house of safety for accident cases. Mercy's best method of giving aid to those who suffer, or are sick in the humbler ranks of society. It diffuses health, and diminishes mortality in large and crowded communities, especially in times of epidemic and fever scares. Its medical school confers inestimable benefits upon every individual member of the community by providing us with the family physician and trained nurse. In fact, the modern hospital has become a great educational institution, where new remedies are tried and tested, where sound hygienic principles are made popular, and where the best and most suitable appliances for the relief of human infirmity and suffering are invented, perfected, and made generally available, to the indescribable benefit and comfort of the human race. Instead of the over-crowded wards and the over-stocked beds, each patient at present has not only a separate bed, but, where the case is exceptionally critical, a separate room also. So far from the patients seeking a refuge from the pestilential atmosphere of the ward by crowding on to a bridge, each ward is provided by modern science with the completest system of ventilation, and in addition the newest hospitals have open verandahs attached to each floor, where surgical cases may have air-baths and sunshine to the utmost possible extent. Sir James Simpson's discovery of chloroform has reduced the shock and suffering of individual patients to a minimum, and our hospital managers have become so humane that the patients are placed under chloroform in a separate room, and are thence wheeled into the operation-theatre in an unconscious condition, from whence they are again removed to their beds, where they awake in comfort, and after undergoing the least possible shock to their nervous systems.

What a contrast, truly, to the Hôtel Dieu of former days! and who that reads will not be grateful for the change, and desirous of supporting the modern system to the fullest extent of his means?

**The Work of the Hospital.** In a special Hospital Sunday and Saturday number of THE HOSPITAL, published on June 4th, the work of our medical institutions in London was set forth in detail, and a statement was made, not only of the work done, but of the money required to accomplish it. In the whole of London proper, containing upwards of four millions of inhabitants, it was shown that one million separate persons received relief at the hospitals in the year 1886; that is to say, that the names of upwards of one million people were entered in the books as new cases receiving medical relief during that year. Of these cases, 60,500 were in-patients, and 943,000 were out-patients. The total expenditure amounted to £533,000, and the total income from charitable and proprietary sources amounted to £390,500, to which £38,000 must be added for patients' payments, making the total income, exclusive of legacies, rather less than £429,000. It will thus be seen that the hospitals spent £103,000 more than they thus received in this one year. But this is not all, for it is well to remind the reader that, despite the establishment of three new special hospitals in London during the present year, such new undertakings are not only inimical to the public interest, but are also quite unnecessary and undesirable. Inimical to the public interest, because every new hospital of this character means a large expenditure of charitable funds upon management expenses, and a diminution in the revenue available for the relief of the really deserving. Unnecessary and undesirable, because the reputable, well-established, and well-managed institutions already in existence contain upwards of 2,100 beds, which are at present unoccupied throughout every day in the year for want of funds. I have said that the expenditure of the London hospitals exceeded the actual income from the sources named in the year 1886 by upwards of £100,000. This deficiency would have been increased by at least another £100,000 had the hospital managers admitted patients to the vacant beds, and kept them constantly occupied throughout the year. It is estimated that one bed to each thousand of the population in rural districts is an adequate proportion, and in crowded cities double that number of beds will meet all requirements. On this basis London ought to contain at least nine thousand hospital beds, and it must be admitted, therefore, that the London hospitals ought to be provided by the people of London with an additional income of at least £200,000 per annum as a mere act of self-protection and prudence on their part.

**The Hospital Sunday Fund.** It has been said over and over again, because, I suppose, the idea is sufficiently taking, and the difficulty of exposing the fallacy of the contention is felt to be great, that the Hospital Sunday Fund has not been an unmixed blessing to the hospitals. Dealing to-day only with the London hospitals, I have to point out that without it much of their work must have been left undone, and many of the institutions themselves would probably never have continued to exist. Hospital Sunday was first systematically organised in London in the year 1876, and if the hospital revenue from charitable sources has steadily increased during the last ten years as compared with the previous ten years, then the institution of Hospital Sunday must be admitted to be helpful to the hospitals individually and collectively. The charitable income of seventy-three London hospitals has increased from £192,736 in 1876 to £256,942 in 1885. That is to say, the free contributions of the people to the London hospitals have increased by rather more than one penny per head per annum of the population during the years Hospital Sunday has been established, whilst the expenditure to meet the needs of the larger population has only increased about one farthing per head. The net charitable revenue of the London hospitals is, therefore, three farthings per head of the population more now than it was when Hospital Sunday was first established, which represents an increase of something like £20,000 a year in the total income of the seventy-three metropolitan hospitals referred to. Those who care to examine further will find all the figures given in detail on page 173, vol. i, of THE HOSPITAL. These figures prove the fallacy of the general statement. Proceeding next from the whole of the hospitals to an individual charity, we find the same evidence of the advantages of the Hospital Sunday Fund proved quite as conclusively. The Royal Kent Dispensary, recently amalgamated with the Miller Hospital, and situated at Greenwich, has stated more than once through its honorary secretary, Mr. Bristow, that the Hospital Sunday Fund has seriously diminished its income. I find, however, on a reference to the report of the Royal Kent Dispensary, that this is not the case. In 1857 the amount received from church collections by this institution was £6 7*s*. There were no other church collections until 1862, when they amounted to £77. The average yearly receipts from church collections for the ten years previous to the establishment of Hospital Sunday were £78, as compared with an average contribution from the Hospital Sunday Fund during the next ten years of £132 per annum. During the last three years the average contributions of the Hospital Sunday Fund to this Dispensary have been upwards of £149, or nearly double the amount received from church collections during the ten years previous to the institution of the Fund. May I hope that this libel, which may be classed with many other popular errors, will not in future be repeated by speakers and preachers, but that they will use these facts to prove what a blessing Canon Miller's Fund has most certainly been to the hospitals and medical charities everywhere?

**Systematic Support.** One of the difficulties of individuals and institutions in London is the vastness of the population and of the acreage contained within the metropolitan area. How can one bring home the facts to individuals, and make every man, woman, and child feel that our hospital system concerns them, and that it is not a mere abstraction, but a real genuine advantage to themselves? Only, I believe, by persistently bringing to the notice of the public the facts about hospitals. These difficulties may account for the fact that whereas in Birmingham seventy contributing congregations collect £5,000 a year for the local hospitals, in London two thousand congregations collect less than £40,000. If the Birmingham average were reached, the collections in London would realize the splendid sum of £150,000 per annum on Hospital Sunday. The hospitals need the systematic support of individuals, rich and poor, prince and peasant, for the monarch on her throne, and the humblest subject of her empire, alike benefit by the hospitals, and ought joyfully to aid to maintain them in adequate numbers and efficiency. There is not a family, high or low, which does not know what sickness and death mean, nor probably many persons who cannot call to mind with thankfulness the happy days of convalescence, and the re-established health which they owe under Providence to the work of the hospitals. As the Duke of Cambridge said last year, it is not the amount of the gift, but the sentiment which inspires the giver, which makes the offering so acceptable and so helpful. Every member of the community, old or young, well-to-do or impoverished, can spare something, if it is only a halfpenny, for the hospitals. We do not desire to have, this year, merely showers of gold, but floods of silver and oceans of coppers, so that the Jubilee Hospital Funds collections, whether they are made in the streets through the instrumentality of the Hospital Saturday boxes, or in the churches and chapels on Hospital Sunday, shall be worthy of every class of the community, and shall collectively represent the great unmistakable outpouring of a grateful people. One hundred thousand pounds at least onght to be raised, not only as a testimony to our gratitude for the great benefits which we have received from the hospitals, but as an adequate guarantee that from this time forth, as prudent men and prudent women, we have determined to provide that adequate means for the relief of sickness and suffering, disease and accident, shall be never wanting to the people of this vast metroplis.

